# A Rare Case of Adult Escherichia coli Meningitis Following Endoscopic Skull-Base Surgery

**DOI:** 10.7759/cureus.92536

**Published:** 2025-09-17

**Authors:** Devi B Yalamanchili, Holly O'Brien, Bushra Shariff, Yilmarie Rosado-Acevedo, Jessica Glover

**Affiliations:** 1 Internal and Hospital Medicine, Moffitt Cancer Center, Tampa, USA; 2 Oncologic Sciences, University of South Florida Morsani College of Medicine, Tampa, USA

**Keywords:** actinomyces odontolyticus, e coli: escherichia coli, e coli meningitis, gram negative meningitis, meningitis, olfactory neuroblastoma, post op meningitis, post surgical meningitis, sinonasal neuroblastoma, skull base tumors

## Abstract

This report describes the case of a 65-year-old male who developed *Escherichia coli (E. coli)* meningitis after endoscopic resection of a sinonasal neuroblastoma, complicated by a cerebrospinal fluid (CSF) leak, pneumocephalus, and subsequent concurrent infection with *Actinomyces odontolyticus*. The patient underwent prolonged, tailored antibiotic therapy and surgical CSF leak repair. The report highlights the importance of suspecting *E. coli* meningitis in post-neurosurgical patients, emphasizes the need for prompt management of CSF leaks, and underscores the need for vigilance regardless of immunocompetency. The patient fortunately survived with a favorable outcome thanks to early diagnosis, immediate intervention, and careful antibiotic management.

## Introduction

While* Escherichia coli (E. coli)* is a common cause of meningitis in the neonatal period, it is exceedingly rare in adults, with fewer than 50 cases documented in the medical literature since 1945 [1,2]. Bacterial meningitis in general has become an uncommon diagnosis in adults in the US, thanks to vaccination, with an estimated incidence of one per 100,000 [3]. Globally, postoperative meningitis occurs in 0.3-25% of cases, with up to 75% being attributable to neurosurgery, neurosurgical devices, and cerebrospinal fluid (CSF) leaks [4-7]. Various studies indicate that 36-50% of gram-negative bacterial meningitis cases occur after neurosurgical procedures [1,4,6]. Risk factors include advanced age, alcohol dependence, liver cirrhosis, diabetes mellitus, and immunocompromised status [4,5,8]. Mortality rates have been reported as 15-34% in bacterial meningitis, but it can be as high as 50-100% in *E.coli *meningitis [1,2,8,9]. Additionally, up to 39% of meningitis patients experience neurological complications [8].

This case report presents a rare instance of adult *E. coli* meningitis following endoscopic resection of a sinonasal neuroblastoma. The objective is to highlight this potentially fatal complication of neurosurgical procedures, particularly with an endonasal approach, to ensure rapid recognition and management. Our patient, fortunately, had a favorable outcome.

## Case presentation

**Figure 1:**
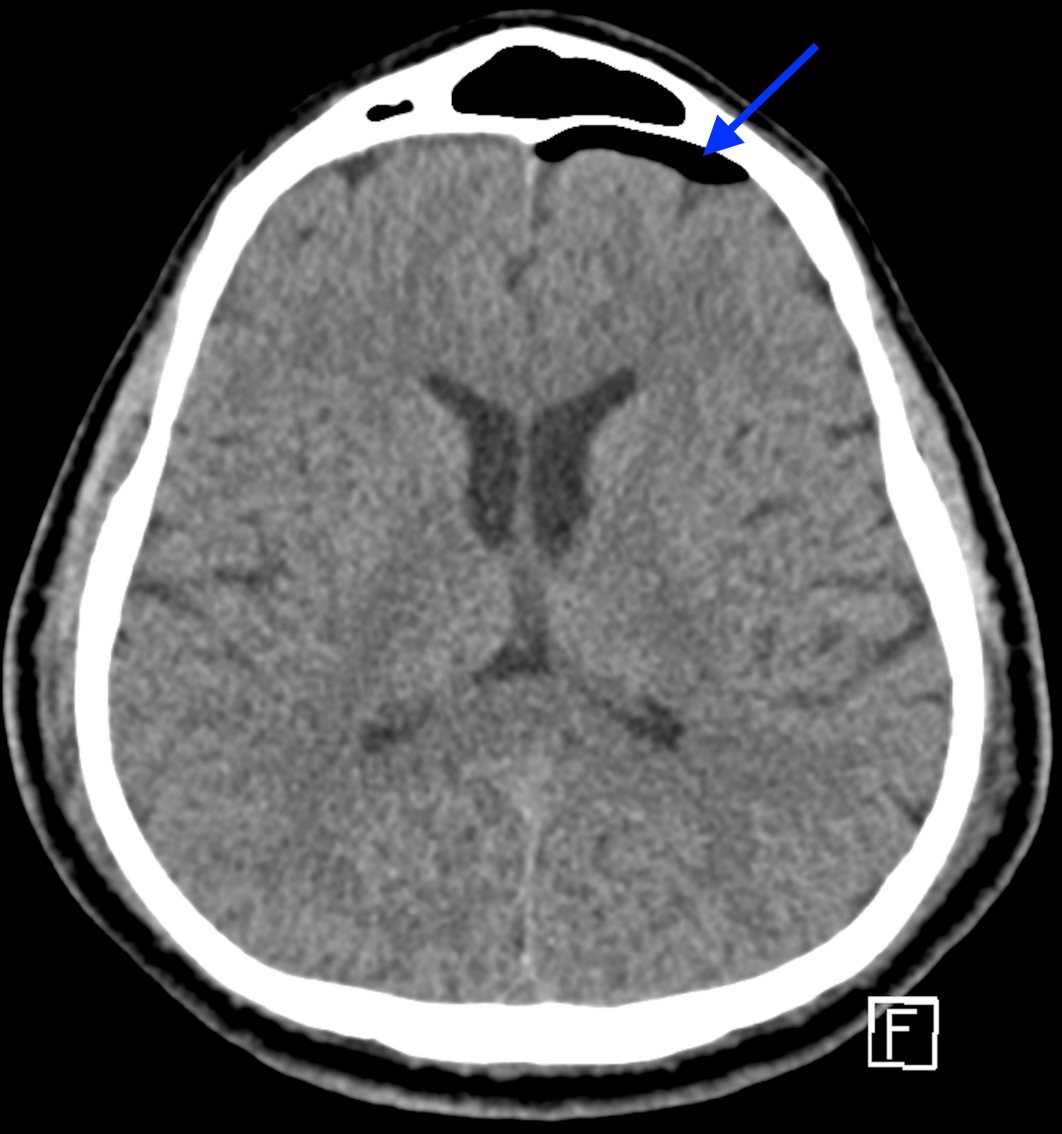
Axial CT head without contrast on postoperative day 2 - image 1 The image demonstrates a new left frontal pneumocephalus (arrow), suggesting CSF leak with air entry CSF: cerebrospinal fluid; CT: computed tomography

**Figure 2:**
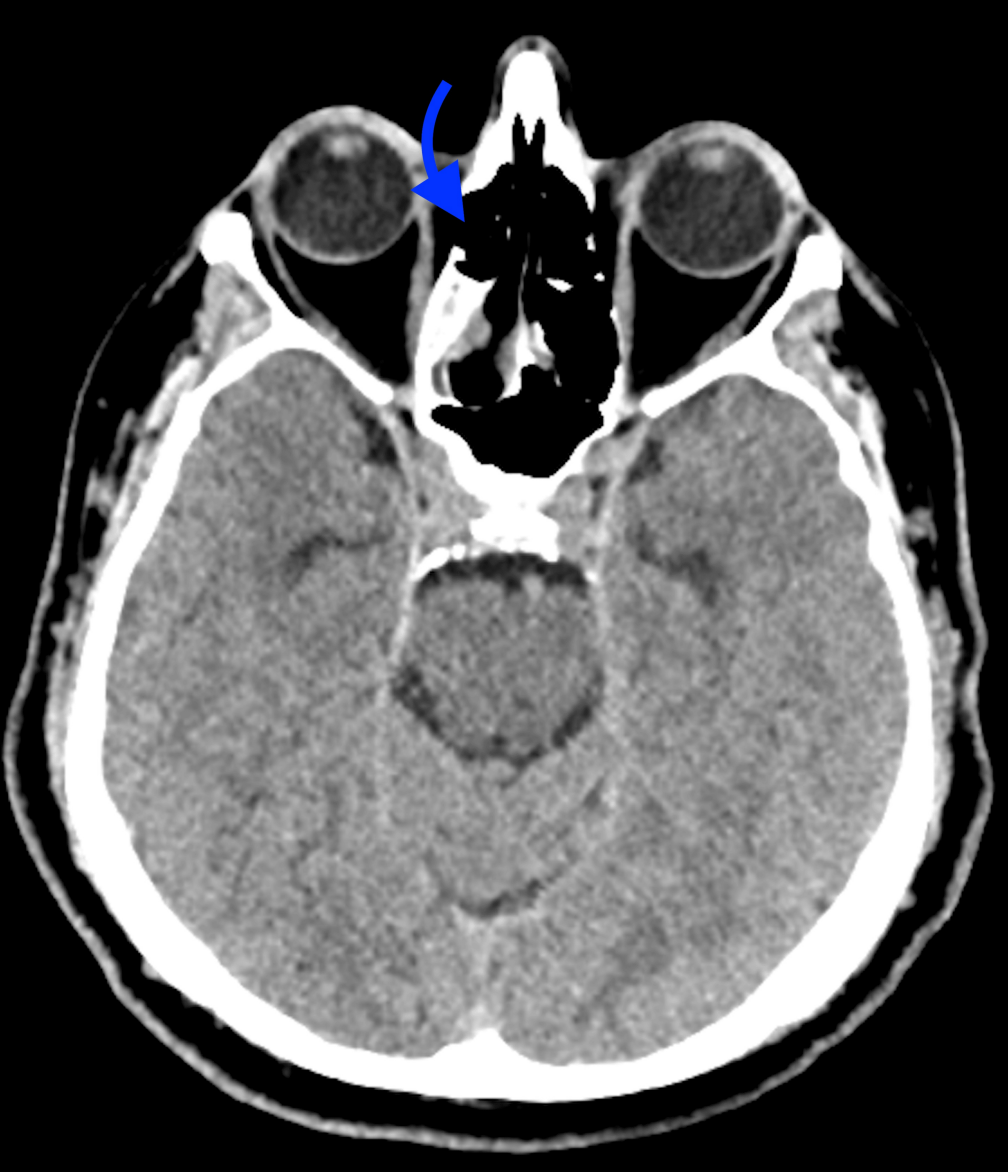
Axial CT head without contrast on postoperative day 2 - image 2 The image demonstrates a right cribriform plate defect after endoscopic sinonasal tumor resection (arrow) CT: computed tomography

A 65-year-old male with a history of well-controlled type 2 diabetes mellitus, olfactory neuroblastoma, and non-alcoholic hepatic steatosis underwent an expanded endoscopic endonasal resection of a right sinonasal neuroblastoma, including nasoseptal flap reconstruction. Two days postoperatively, clear nasal drainage suspicious for a CSF leak was noted, and beta-2 transferrin and stat CT head without contrast were ordered. Unfortunately, the beta-2 transferrin was cancelled by the laboratory and is therefore unavailable for review. Given the persistent, clear nasal drainage on examination and associated new left frontal pneumocephalus on imaging (Figure 1), he was treated empirically for a CSF leak with prompt lumbar drain placement and acetazolamide initiation. CT head also showed a right cribriform defect post-craniectomy and tumor resection (Figure 2).

That evening, the patient developed fever (38.4°C), lethargy, and headache. Concern for meningitis led to empiric treatment with vancomycin, cefepime, and acyclovir, with critical care consultation. Repeat stat CT head without contrast demonstrated a stable right cribriform defect and left frontal pneumocephalus. A lumbar puncture revealed white blood cells of 4250/mm³, red blood cells of 900/mm³, 99% neutrophils, protein of 1142 mg/dL, and glucose of 71 mg/dL. CSF gram stain revealed gram-negative rods, subsequently identified as *E. coli*, which was found to be pan-sensitive on susceptibility testing. Infectious Disease was consulted to assist with antibiotic management. Antibiotics were de-escalated to ceftriaxone 2g IV every 12 hours, and acyclovir was discontinued following a negative CSF HSV PCR. The patient was additionally treated with dexamethasone 4 mg every six hours for vasogenic edema and levetiracetam 500 mg every 12 hours for seizure prophylaxis. His headache and delirium significantly improved, and nasal drainage resolved.

**Figure 3:**
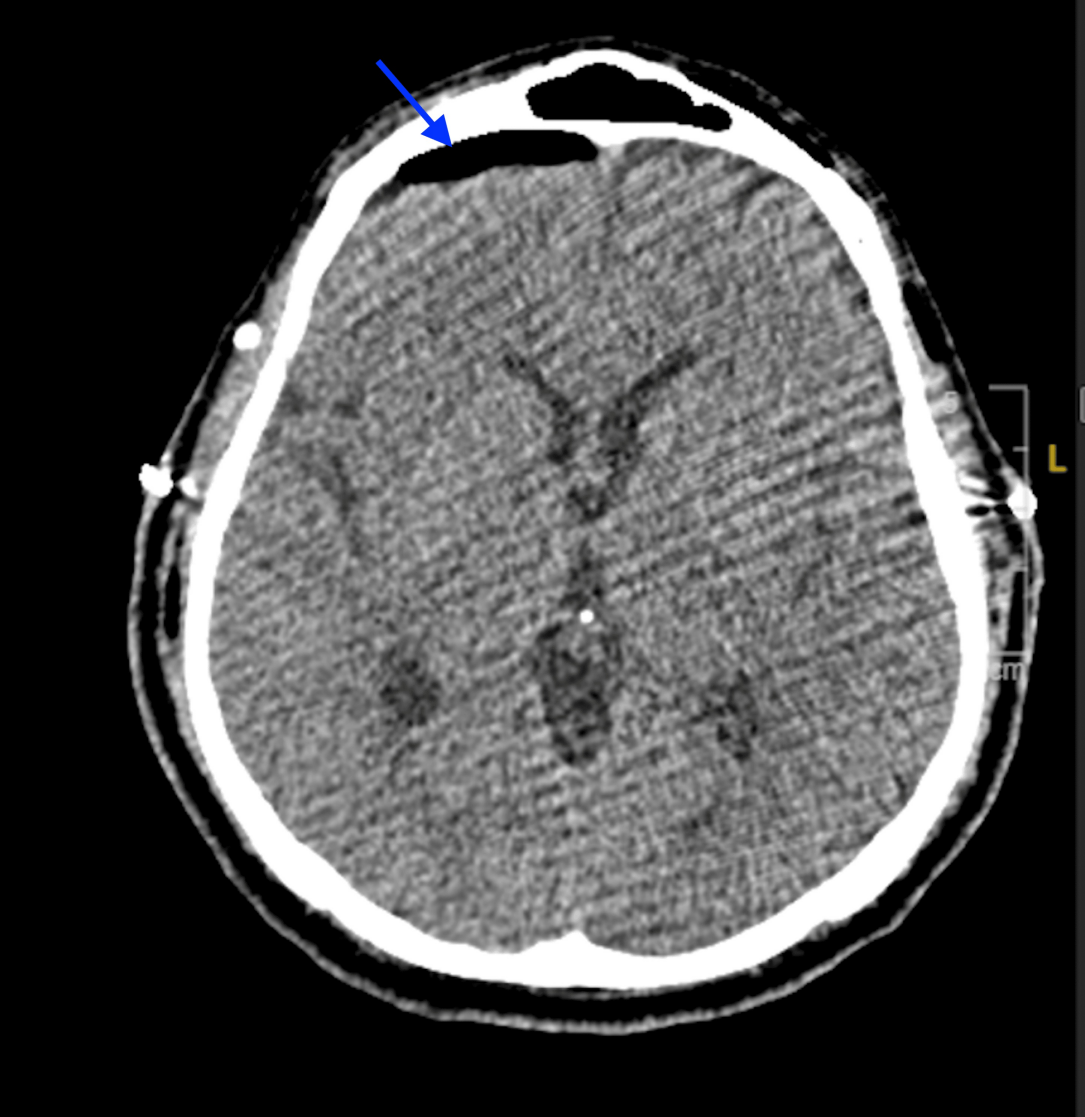
Axial CT head without contrast on postoperative day 4 The image demonstrates a new right frontal pneumocephalus (arrow), suggesting CSF leak with air entry CSF: cerebrospinal fluid; CT: computed tomography

On postoperative day four, the patient experienced a severe headache, prompting another stat CT head without contrast that revealed resolution of the prior left frontal pneumocephalus but new right frontal pneumocephalus, suggesting a significant CSF leak with air entry (Figure 3). Consequently, the lumbar drain was removed, and the following day, he underwent the placement of a new lumbar drain and pericranial flap repair of the CSF leak. The antibiotic course was adjusted with a plan for an additional 14 days following the most recent surgical intervention, contingent on CSF leak resolution. The lumbar drain was maintained with continuous fluid removal at 15 ml/hour. High-dose steroids for vasogenic edema and levetiracetam for seizure prophylaxis were continued.

The CSF thioglycolate broth culture from postoperative day four subsequently returned positive for *Actinomyces odontolyticus*. Although the possibility of contamination was considered, the sinonasal surgical approach provided a potential direct pathway for this organism, which is commonly found as part of the oral flora and typically encountered in polymicrobial infections. Consequently, the infectious disease specialist recommended managing it as a true co-infection. Ceftriaxone covered both the *E.coli *and *Actinomyces odontolyticus* during hospitalization. The lumbar drain was clamped on postoperative day four, status post-drain replacement, and removed on postoperative day five after stable follow-up imaging.

Subsequently, the patient developed thrombocytopenia, thought to be due to the antibiotic regimen. Ceftriaxone was switched to ciprofloxacin as a precaution, despite only a rare association with drug-induced thrombocytopenia, with Neurosurgery opting to continue levetiracetam. Amoxicillin was initiated for the *Actinomyces odontolyticus*. He was discharged home on a 14-day course of ciprofloxacin 500 mg every 12 hours and a planned 12-month course of amoxicillin 1 g every eight hours with close outpatient follow-up. At the six-month follow-up visit with Infectious Disease, a review of post-discharge nasal endoscopy from the most recent ENT visit a month prior demonstrated stable findings, and the patient was noted to have clinically improved.

## Discussion

While gram-negative meningitis is rare in non-surgical patients, it accounted for approximately half of post-neurosurgical bacterial meningitis cases in a retrospective analysis by Zeinalizadeh et al. [6]. Several factors elevated this patient’s risk, including tumor resection, diabetes, a procedure crossing the sinus (a high-risk area of bacterial colonization), and a CSF leak [4,7]. The CSF leak, which presented two days postoperatively, may have preceded or precipitated the fulminant meningitis, and the endonasal exposure further increased this patient’s risk. Reports show CSF leak rates for anterior skull base malignancies generally range from 5-10% [10]. A meta-analysis and systematic review by Kim and Hong found that the use of a pedicled vascularized flap decreases this risk, while the use of a lumbar drain had conflicting data [10]. A pedicled vascularized flap was utilized in this patient, though a lumbar drain was not placed until evidence of CSF leak was noted.

While *E. coli* colonization of the sinuses is unusual, the subsequent *Actinomyces odontolyticus* is more consistent with translocation of sinonasal or oral flora [6]. As seen in our case, the *Actinomyces *growth in the thioglycolate broth complicated the clinical picture with concern for contamination, but the clinical context of the sinonasal surgical approach supported the likelihood that the *Actinomyces* was a true co-infection. Urine and blood cultures were negative, and no other focus of infection was identified. *Actinomyces* is also a rare CNS pathogen, with pachymeningitis reported in only nine cases [11]. It remains endogenous to the oral and gastrointestinal mucosa but may become pathogenic to cause an inflammatory response when the mucosal barrier is compromised. A prolonged course of antibiotics is generally necessary for CNS involvement by *Actinomyces*, initially with IV ceftriaxone or penicillin, and subsequent transition to oral antibiotics [12]. Duration of treatment for moderate to severe infections involving *Actinomyces* can range from six to 12 months or longer, depending on radiographic and clinical response [12].

In this case, rapid recognition, diagnosis, and management were facilitated by the patient’s hospitalization and the availability of infectious disease subspecialists. When encountering such a presentation, it is important to note that older patients (≥65 years) with meningitis are more prone to atypical presentations such as coma, seizures, or hemiparesis, and may lack classic signs like fever, nuchal rigidity, headache, or vomiting [8]. This patient had fever, headache, and altered mental status, but not the full classic triad (i.e., fever, headache, nuchal rigidity) traditionally characteristic of meningitis. However, the patient’s presentation may be typical for post-neurosurgical patients [6]. The retrospective analysis by Zeinalizadeh et al. found that fever, headache, and altered mentation are, in that order, the most common presenting symptoms of post-craniotomy meningitis [6].

Lumbar puncture with CSF analysis remains essential for diagnosing meningitis, ideally performed before antibiotic administration to avoid false-negative results [8,9]. However, rapid antibiotic administration is critical for reducing mortality and should not be delayed for lumbar puncture [8,9]. Pre-lumbar puncture CT head evaluation is a critical step in patients with altered mental status, neurological deficits, new-onset seizures, or a history of intracranial lesions to identify cerebral edema or mass effect that could increase the risk of herniation during lumbar puncture [8,9].

A review of the literature revealed fewer than 50 reported cases of adult *E. coli* meningitis [1,2]. Risk factors cited include alcohol dependence with liver cirrhosis, HIV, organ dysfunction, uncontrolled diabetes, disseminated strongyloidiasis, and neurosurgery [1,2,5]. Neurosurgery is a significant risk factor, though meningitis affects fewer than 5% post-craniotomy [7]. Specifically, tumor resection, procedures near areas of high bacterial colonization (e.g., paranasal sinuses), CSF leaks, and concurrent non-CNS infections are associated with postoperative infections [7]. Catheters, including CSF shunts and external ventricular drains, also contribute to risk [7]. In this case, the surgical intervention traversing the cribriform plate, combined with the CSF leak, was likely the most probable etiology. Preventative strategies, such as the use of a vascularized flap, need to be emphasized [10]. Timely diagnosis and appropriate management are crucial due to the high mortality of 50-100% and risk of long-term neurological consequences associated with *E. coli* meningitis [1,2,8,9]. Raising awareness of this diagnosis is essential, particularly as the incidence of neurosurgical procedures and bacterial resistance is on the rise.

## Conclusions

This report highlights a rare but serious post-neurosurgical complication: *E.* *coli* meningitis in an adult patient following endonasal surgery with a delayed CSF leak. Early recognition, aggressive surgical management of leaks, and prolonged, targeted antibiotic therapy were crucial for a successful outcome. Greater emphasis and further investigation are warranted regarding strategies to prevent postoperative CSF leaks and meningitis. Clinicians should maintain a high index of suspicion for this pathogen even in patients without classic immunosuppression (e.g., HIV infection, chemotherapy, transplant) who undergo procedures involving skull-base exposure.
